# Determinants of abortion among clients coming for abortion service at felegehiwot referral hospital, northwest Ethiopia: a case control study

**DOI:** 10.1186/s40834-017-0038-5

**Published:** 2017-02-14

**Authors:** Fikreselassie Tilahun, Abel Fekadu Dadi, Getachew Shiferaw

**Affiliations:** 1 Debrebrhan Hospital, Debre Berhan, Ethiopia; 2 Institute of Public Health, College of Medicine and Health Sciences, University of Gondar, P.O.Box: 360, Gondar, Ethiopia; 3 College of Medicine and Health Sciences, University of Gondar Hospital, Gondar, Ethiopia

**Keywords:** Case-control study, Induced abortion, Ethiopia

## Abstract

**Background:**

According to the World Health Organization (WHO) estimate, one-third of pregnancies end in miscarriage, stillbirth, or induced abortion in the world. There are various reasons for a woman to seek induced abortion. However, limited information is available so far in the country and particularly in the study area. Therefore, the aim of the current study was to identify the determinants of induced abortion among clients coming for abortion care services at Bahirdar Felegehiwote referral hospital, Northwest Ethiopia.

**Methods:**

Institutional based unmatched case-control study was conducted from September to December 2014. Interview administered questioner was used to collect primary data. Enumeration and systematic random sampling (K = 3) method was used to select 175 cases and 350 controls. A binary logistic regression model was fitted to identify determinant factors. Odds ratio with 95% CI was computed to assess the strength and significance of the association.

**Result:**

All sampled cases and controls were actually interviewed. The likelihood of abortion was higher among non-married women [AOR: 18.23, 95% CI: 8.04, 41.32], students [AOR: 11.46, 95% CI: 6.29, 20.87], and women having a monthly income of less than 500 ETB [AOR: 11.46, 95% CI: 6.29, 20.87]. However, the likelihood of abortion was lower among women age greater than 24 years [AOR: 0.29, 95% CI: 0.11, 0.79] and who had the previous history of induced abortion [AOR: 0.31, 95% CI: 0.15, 0.65].

**Conclusion:**

The study identified being non-married, student, women age less than 24 years, having the previous history of induced abortion, and low monthly income as an independent determinant of induced abortion. Interventions focused on the identified determinant factors are recommended.

## Background

Every day, approximately 1000 women die from preventable causes related to pregnancy and childbirth worldwide and 99% of all maternal deaths occur in developing countries [1]. World Health Organization (WHO) estimates that, worldwide 210 million women become pregnant each year and about two-thirds of them, or approximately 130 million, deliver live infants [1, 2]. The remaining one-third of pregnancies ends in miscarriage, stillbirth, or induced abortion [3]. Of the estimated 42 million induced abortions each year, nearly 20 million are performed in unsafe conditions and results in the deaths of an estimated 47,000 women. This represents about 13% of all pregnancy-related deaths [3, 4].

Generally, the burden of abortion is still significant being one of the leading causes of maternal mortality and morbidity. An estimated 68,000 girls and women die of unsafe abortion and millions are injured [5, 6]. The ratio of abortion deaths per 100,000 procedures is less than 1/100,000 in developed countries while it is 680/100,000 for Africa [7–9]. This category of abortion was even called “silent scourge” because of the high number of deaths attributable to it and its economic and social consequences [10].

The maternal mortality ratio (MMR) in Ethiopia was estimated as 420 deaths per 100,000 live births in the year 2015 [11]. According to the World Health Organization, Ethiopia had the fifth largest number of maternal deaths in 2005 and unsafe abortion is estimated to account for 32% of all maternal deaths in Ethiopia [12]. It is estimated that there are 3.27 million pregnancies in Ethiopia every year, of which approximately 500,000 ends in either spontaneous or induced abortion [13].

In 2005 Ethiopia expanded its abortion law that had previously allowed the procedure only to save the life of a woman or protect her physical health. Currently, abortion is legal in Ethiopia under certain preconditions like in cases of rape, incest or fetal impairment, if the pregnancy endangers her or her child’s life, or if continuing the pregnancy or giving birth endangers her life. A woman may also terminate a pregnancy if she is unable to bring up the child, owing to her status as a minor or to a physical or mental infirmity [14].

Reasons of women to seek induced abortion are different as to the various women circumstances. A review from 27 different countries revealed that postponing, stopping childbearing and socio-economic factors such as being unable to afford a child- either in terms of a direct cost of raising a child or opportunity costs are the commonest reasons for induced abortion [15].

A study conducted in Denmark and Uganda identified being single followed by being aged 19 years or below, having two children or more, student or unemployed as the strongest determinant of women’s decision to have an abortion [16, 17]. In Ethiopia, similar findings were revealed that economic problem, family size control, being single, the level of education attained by the mother, and extra-marital pregnancy were the factors that increase induced abortion [18–20].

Having this much significance of the problem, little is known about the reasons that drive women to terminate their pregnancy. Therefore, this study was conducted to identify the determinants of induced abortion. The current study was different from previous in design, and the study site, which was regional referral hospital that includes diverse population. This could support the government efforts made to prevent induced abortion and its consequence.

## Method

### Study setting and population

The study area was Felegehiwot referral hospital, which is found in Bahirdar city administration, Northwest of Ethiopia. Bahir Dar is found at a distance of 563 km away from Addis Ababa and located on the Southern shore of Lake Tana, the source of the Blue Nile. In Bahir Dar city administration, for the total of 31,800 populations, there are 2 hospitals, 10 health centers, 10 health posts, and 134 private health institutions. Felegehiwot referral hospital is one of the other referral hospitals found in the country, in which the services given for clients and the clients coming to the hospital are comparable with other similar hospitals. Researchers employed a hospital-based unmatched case-control study from September to December 2014. The source and study population consisted of pregnant women seeking maternal care services at maternal care ward. Seriously ill mothers and mothers with spontaneous abortion were excluded from the study.

- *Cases* were selected from reproductive age women for whom safe abortion was performed or for whom post-abortion care service was provided after presenting to the hospital with an attempt of induced abortion within 1 week of presentation.
- *Controls* were selected from antenatal care attendees in Felegehiwot referral hospital who persisted on their pregnancy after the 28^th^ completed gestational age.

### Sampling technique and sample size determination

A systematic random sampling (K = 3) was used to select controls and enumeration method was used to include cases. The first interviewer in the case of control selection was based on lottery method. Researchers calculated the sample size using the Epi Info 7 STAT CALC program by taking assumptions of a 95% confidence level, 80% power and a number of children the mother had as a primary exposure variable [18]. Fifteen (15.8%) of control women had two or more children and, 27.3% of women with induced abortion had two or more children (OR = 2). Researchers used two controls for each case and the final sample size for the study adding 10% non-response rate was 175 cases and 350 controls.

### Data collection and quality assurance

Pregnant women who were available during data collection period were eligible to participate in the study. Researchers used a pretested and structured Amharic questionnaire to collect information. Face-to-face interviews were conducted to collect data on independent and socio-demographic variables. Language experts translated the questionnaire from Amharic to English and back to English to ensure consistency. Researchers conducted pretest on the different area by taking 10% of the total sample size and used the revised questioner for final data collection. Two midwifery nurses collected the data after being trained. The principal investigator critically supervised the whole process of the data collection. Knowledge about emergency contraceptive was assessed by a single question and they were considered knowledgeable if they have answered the question as yes. However, mothers were asked to mention types of contraceptive methods and their knowledge was categorized as “mentioned at least one type” and “mentioned more than one type” based on their response.

### Data analysis

All the data were checked, coded and entered using Epi Info version 7 and analyzed using STATA Version 12. Researchers checked the extent of outliers, the different statistical assumptions, and applied the appropriate correction mechanisms prior to analysis. Association of each independent variable was assessed with binary logistic regression. Variables showing statistically significant associations with the outcome variables (up to *p* = 0.2) were considered as potential confounders of abortion and simultaneously subjected to stepwise multiple logistic regression models to determine the significant independent risk factor of abortion. Adjusted odds ratio with a *p*-value < 0.05 was used to report the significant factors.

## Result

### Socio-demographic characteristics of the mothers

All case and control women expected were interviewed. Among interviewed women, 78 (44.6%) of cases and 235 (67.1%) of controls were urban residents. One hundred thirty-two (75.4%) of the cases and 131 (37.4%) of the controls were less than 24 years and about 150 (85.7%) of cases and 305 (87.1%) of controls were Orthodox Christianity followers. Regarding the educational status of the women, 64 (36.5%) of the cases and 136 (38.8%) of the controls were below secondary school while the rest were a secondary and above level of education (Table 1).

**Table 1:** Socio- demographic characteristics of mothers came to Felege Hiywot Referral Hospital, Amhara Region, North West Ethiopia

Study variables	Abortion status	
	Case, *N* (%)	Control, *N* (%)
Residence		
Urban	78 (44.6)	235 (67.1)
Rural	97 (55.4)	115 (32.9)
Age of the mother		
< 24 years	132 (75.4)	131 (37.4)
> =24 years	43 (24.6)	219 (62.6)
Religion		
Orthodox	150 (85.7)	305 (87.1)
Other religion followers	25 (14.3)	45 (12.9)
Educational status of the mother		
Below secondary school	64 (36.5)	136 (38.8)
Secondary and above	111 (63.5)	214 (61.2)
Occupation		
Student	84 (48)	11 (3.1)
Non-student	91 (52)	339 (96.9)
Marital status		
Single	103 (58.8)	31 (8.9)
Married	72 (41.2)	319 (91.1)
Income		
< =500 ETB	105 (60.0)	48 (13.7)
> 500 ETB	70 (40.0)	302 (86.3)

### Maternal characteristics of the women

Concerning family planning usage of the women, 55 (31.4%) of cases and 77 (22%) of controls had not ever used any method of family planning. Ninety-six (54.8%) of cases and 143 (40.8%) of controls had knowledge about emergency contraceptive method while 50 (28.6%) of cases and 67 (19.1%) of controls had ever used an emergency contraceptive (Table 2).

**Table 2:** Maternal characteristics of mothers came to Felege Hiywot Referral Hospital, Amhara Region, North West Ethiopia

Study variables	Abortion status	
	Case, *N* (%)	Control, *N* (%)
Family planning method used		
Ever used no method	55 (31.4)	77 (22.0)
Ever used at least one method	120 (68.6)	273 (88.0)
Emergency contraceptive knowledge		
Yes	96 (54.8)	143 (40.8)
No	79 (45.2)	207 (59.2)
Emergency contraceptive method used		
Yes	50 (28.6)	67 (19.1)
No	125 (71.4)	283 (80.9)
Number of live children the mother had		
Had no children	122 (69.7)	185 (52.8)
Had at least one children	53 (30.3)	165 (47.2)
Previous abortion history		
Yes	15 (8.6)	57 (16.3)
No	160 (91.4)	293 (83.7)
Contraceptive method knowledge		
Mention at least one method	122 (69.7)	348 (99.4)
Mention no method	53 (31.3)	2 (0.6)
Gravidity		
One pregnancy	122 (69.7)	133 (38.8)
Two and above pregnancy	53 (31.3)	217 (61.2)

### Reason for requesting abortion

The reason for requesting abortion was collected from cases of the current study. Accordingly, from 175 cases, the majority 63 (36%) mentioned that the reason for their request was fear of school dropout. The other reasons that frequently mentioned by the cases were maternal related problems followed by unwanted pregnancy.

### Determinants of Mothers’ abortion

After adjusting for other confounding variables: age and occupation of the mothers, marital status, monthly income, and mother’s previous abortion history were the variables that retained in the final model.

Likewise, the odd of abortion among mothers of age 24 and above years was 0.31 [95% CI: 0.15, 0.65]. Similarly, the odd of abortion among the students was 7.4 [95% CI: 2.93, 18.69] times higher as compared to their counterparts. Marital status of the mothers was also the other variable that was significantly associated with mother’s choice to undertake abortion. Likewise, non-married (single) were 18.23 [95% CI: 8.04, 41.32] times more likely to abort as compared to married mothers. Similarly, the odds of abortion was 11.46 [95% CI: 6.29, 20.87] times higher among mothers of monthly income less than or equal to five hundred Ethiopian birr.

Among factors related to maternal characteristics, mothers’ previous abortion history was the only factor that significantly affected mother’s decision to commit abortion. Correspondingly, the odds of committing current abortion was 0.29 [95% CI: 0.11, 0.79] times lesser among mothers who reported previously history of abortion (Table 3).

**Table 3:** Factors associated with requesting abortion of mothers came to Felege Hiywot Referral Hospital, Amhara Region, North West Ethiopia

Study variables	Abortion status			
	Case, *N* (%)	Control, *N* (%)	COR (95% CI)	AOR (95% CI)
Age of the mother				
< 24 years	132 (75.4)	131 (37.4)	1	1
> =24 years	43 (24.6)	219 (62.6)	0.19 (0.13, 0.29)	0.31 (0.15,0.65)*
Occupation				
Student	84 (48)	11 (3.1)	28.44 (14.56,55.57)*	7.40 (2.93,18.69)*
Non student	91 (52)	339 (96.9)	1	1
Marital status				
Single	103 (58.8)	31 (8.9)	14.72 (9.14,23.69)*	18.23 (8.04,41.32)*
Married	72 (41.2)	319 (91.1)	1	1
Income				
< =500 ETB	105 (60.0)	48 (13.7)	9.43 (6.14,14.49)*	11.46 (6.29,20.87)*
> 500 ETB	70 (40.0)	302 (86.3)	1	1
Family planning method used				
Ever used no method	55 (31.4)	77 (22.0)	1.62 (1.08,2.44)*	
Ever used at least one method	120 (68.6)	273 (88.0)	1	
Emergency contraceptive knowledge				
Yes	96 (54.8)	143 (40.8)	1.76 (1.22,2.54)*	
No	79 (45.2)	207 (59.2)	1	
Ever used Emergency contraceptive				
Yes	50 (28.6)	67 (19.1)	1.69 (1.11,2.58)*	
No	125 (71.4)	283 (80.9)	1	
Number of live children the mother had				
No children	122 (69.7)	185 (52.8)	1	
One child and above	53 (30.3)	165 (47.2)	0.49 (0.33,0.72)	
Previous abortion history				
Yes	15 (8.6)	57 (16.3)	0.48 (0.26,0.88)	0.29 (0.11,0.79)*
No	160 (91.4)	293 (83.7)	1	1
Gravidity				
One pregnancy	122 (69.7)	133 (38.8)	1	
Two and above pregnancy	53 (31.3)	217 (61.2)	3.75 (2.54,5.53)*	

*significant at *P*-value < 0.05

## Discussion

The current case-control study was conducted to identify factors that determine mothers’ abortion request. Accordingly, age, occupation, marital condition, and income of the mothers were identified as socio-demographic determinant factors while previous abortion history was the only factor that was related to maternal characteristics.

Age of the cases as a factor for requesting abortion is repeatedly reported in different studies conducted so far [16, 17]. Similarly, in this study, 75.4% of cases requesting abortion are those aged less than 24 years. This might be due to the fact that most of the time females in this age category were in schools or they were not economically and socially able to lead their life.

The alarming finding of this study was that, majority of the cases were students and they were seven times higher to commit abortion compared to their controls. This was revealed by different studies conducted so far in which considerably high percent of cases were students [15, 21–23]. Moreover, from the descriptive data obtained, 36% of the respondents mentioned that their reason to request abortion was fear of school dropout.

The other socio-demographic variable which had an effect on mothers’ decision to request an abortion was marital status. The study revealed that being non-married (single) puts the cases on eighteen times higher risk of requesting abortion compared to the married ones. This finding was also supported by studies published elsewhere [18, 19]. This could also be because of economic and social factors related to the mother and psychological impact related to the newborn while growing in the environment of missing fathers. Moreover, mothers could fear to bring up their newborn independently in such environment.

The other important factor that was associated with higher proportion of cases was their lower economic background. The odds of requesting abortion was eleven times higher among cases who reported that their monthly income was less than five hundred Ethiopian birr. This finding was comparable with a review from 27 different countries and similar studies conducted in Ethiopia [15, 20]. The possible explanation might be women with the lower economic condition might face a challenge to care and grow their child. Besides, 2.3% of the mothers mentioned that the reason for requesting the current abortion was the presence of too many children in the household and this might pose an additional challenge to the family.

From maternal characteristics, previous abortion history was the only factor that had a significant association with mothers’ tendency to request the current abortion. Likewise, the odd of requesting the current abortion was 71% lesser among women having previous abortion history compared to their foil and it is in agreement with previously published articles [24, 25]. This might be the result of a positive lesson learned from the previous abortion. Furthermore, 2.3% of the mothers mentioned that fear of the previous complication made them abort the current pregnancy.

The study employed relatively sufficient sample size that could enhance the representativeness of the population. Incident cases were used to minimize the problem of establishing a temporal relationship and as the study was conducted at regional referral hospital with an experienced gynecologist, the issue of misclassification between spontaneous and induced abortion could be resolved. However, since the study was an institutional based case-control study, other limitations of such study might not be avoided and the study finding is also prone for social desirability bias.

## Conclusion

A considerable proportion of abortion was significantly higher among cases with age less than 24 years, students, unmarried, had no previous abortion history, and lower monthly income of 500 Ethiopian birr. Therefore, Bahirdar health office and regional health bureau shall take intervention targeting the identified factors could probably reduce the burden of induced abortion and its consequences in this population.
